# Short-Term Within-Host Genomic Diversity and Clone Turnover of Carbapenem-Resistant *Klebsiella pneumoniae* in an Intensive Care Unit Patient

**DOI:** 10.3390/antibiotics15060605

**Published:** 2026-06-14

**Authors:** Yulia Mikhaylova, Anna Slavokhotova, Oksana Ni, Denis Protsenko, Sergey Bruskin, Andrey Shelenkov, Vasiliy Akimkin

**Affiliations:** 1 Central Research Institute of Epidemiology, Novogireevskaya Str., 3a, 111123 Moscow, Russiavgakimkin@yandex.ru (V.A.); 2 Moscow Multidisciplinary Clinical Center «”Kommunarka“, Sosenskiy Stan Str., 8c3, 108814 Moscow, Russia; 3 Vavilov Institute of General Genetics, Russian Academy of Sciences, Gubkina Str., 3, 119991 Moscow, Russia

**Keywords:** ESKAPE pathogens, carbapenem resistance, within-host evolution, clonal diversity, ICU-acquired infection, plasmid dynamics, genomic epidemiology, whole-genome sequencing

## Abstract

**Background**: Carbapenem-resistant *Klebsiella pneumoniae* (CRKP) is a critical public health threat because infections caused by this pathogen are associated with high morbidity, mortality, and limited effective therapeutic options. Whilst the majority of studies have concentrated on inter-patient bacterial transmission, within-host genomic analysis offers unprecedented resolution for tracking dynamic clone predominance, plasmid rearrangements, and microevolution under clinical selection pressures. **Methods and Results**: Whole-genome sequencing (WGS) of nine isolates recovered from oral and rectal swabs revealed an exceptional case of CRKP clonal turnover in an intensive care unit (ICU) patient. Three distinct high-risk clones were identified during the 18 days of surveillance: an initial ST101 (Clonal Group (CG) 101) strain (days 1–7) followed by concurrent colonization with ST395 (carrying *bla*_NDM-5_) and ST512 lineages (both CG258, days 11–18). **Conclusions**: This study describes a rare instance of within-host heterogeneity of CRKP, involving three distinct STs spanning two CGs. Whole-genome analysis revealed potential structural rearrangements of resistance- and virulence-associated plasmids between coexisting lineages. These genomic shifts likely reflect rapid adaptation under the intense selective pressure of broad-spectrum antibiotic therapy, culminating in the persistence of a less virulent yet multidrug-resistant ST512 clone and a favorable clinical outcome with patient recovery.

## 1. Introduction

The rapid spread of Enterobacterales resistant to carbapenems, the antibiotics of last resort, made the World Health Organization include these bacteria in a critical priority group for research and development of novel antibiotics [1]. *Klebsiella pneumoniae*, a member of the *Enterobacteriaceae* family belonging to Enterobacterales order, is one of the most prominent bacterial pathogens causing nosocomial and opportunistic infections of the respiratory system, urogenital tract and bloodstream [2]. Due to its exceptional genome plasticity and capacity to assimilate diverse genetic elements, *K. pneumoniae* has emerged as a major reservoir and disseminator of antibiotic resistance determinants across bacterial populations [3,4]. Carbapenem resistance in *K. pneumoniae* (CRKP) is predominantly mediated by carbapenemase production and reduced permeability of the cell wall membrane [5,6]. The particular carbapenemase gene families, *bla*_KPC_, *bla*_NDM_, *bla*_VIM_, and *bla*_OXA-48_ are of major concern because they usually reside on mobile genetic elements—such as plasmids, transposons, and integrons—that facilitate their rapid dissemination through horizontal gene transfer [7].

The *K. pneumoniae* population consists of two divergent branches: classical (cKp) and hypervirulent (hvKp) pathotypes. CKp is considered a part of the opportunistic microbiota present in healthy individuals and is also associated with hospital-acquired pneumonia [8]. Although globally disseminated, this pathotype exhibits relatively restricted virulence. However, it readily acquires diverse mobile genetic elements through horizontal gene transfer, generating multidrug-resistant (MDR) strains that are frequently incorporated into high-risk clones. Multilocus sequence typing (MLST) has identified several high-risk clones of *K. pneumoniae*, with ST11, ST14, ST15, ST17, ST45, ST147, ST258, ST307, ST395 and ST512 being the most well-studied and clinically significant [9,10,11].

The hvKp is distinguished from cKp by significantly enhanced pathogenicity, conferred by a constellation of virulence factors. These include a hypercapsule producing the characteristic hypermucoid phenotype, multiple siderophores facilitating iron acquisition, an allantoin utilization system, colibactin toxin, and type I fimbriae enabling bacterial adhesion to host tissues [3,12].

Such strains cause aggressive community-onset infections in healthy individuals and are mainly represented by ST23-K2, ST65, and ST86 clones. It is noteworthy that, until recently, MDR genetic lineages were largely restricted as cKp; however, emerging reports from multiple countries now document the appearance of MDR clones within hvKp populations as well [2,13,14]. Furthermore, hvKp can acquire additional mobile genetic elements carrying carbapenemase genes via horizontal gene transfer. Consequently, the previously theoretical concerns regarding the emergence of a “superpathogen” combining hypervirulence with multidrug resistance, including resistance to carbapenems, have now materialized as a clinically significant reality [2,15,16,17].

In hospital conditions, rectal colonization of patients with CRKP is recognized as a significant risk factor for healthcare-associated infections [18]. Extraintestinal infections (septicemia and pneumonia in most cases) following CRKP colonization were associated with increased risk of transmission between patients, morbidity, mortality, progression of infection and prolonged hospital stay [19,20].

These findings collectively demonstrate the urgent need for enhanced genomic surveillance of carbapenem-resistant isolate distribution, particularly VIM/KPC/NDM/OXA-48-carrying isolates. Notably, hospital-based studies frequently lack comprehensive molecular epidemiological characterization, particularly with regard to bacterial strain persistence and the acquisition and emergence of key genetic determinants conferring resistance mechanisms, which remain inadequately investigated. In contrast, longitudinal intra-patient genomic surveillance enables the detection of de novo mutations in resistance genes and plasmid that emerge during protracted chronic infections and/or prolonged antimicrobial therapy. In this study, we characterized the genomic diversity and clonal turnover of CRKP isolates collected from serial oral and rectal swabs of a single patient during three-week intensive care unit (ICU) admission using whole-genome sequencing (WGS) and comprehensive bioinformatics analysis.

## 2. Results

### 2.1. Genome Assembly, Typing and Phylogenetic Analysis

The characteristics of genome assemblies and typing results for the isolates studied are provided in Table 1.

In silico MLST revealed that nine *K. pneumoniae* isolates were divided into three genetic lineages: ST101 (belonging to Clonal Group (CG) 101), ST395 (CG258) and ST512 (CG258). It is worth noting that the identified lineages belong to the “high-risk clonal groups” of *K. pneumoniae* [11]. Three ST101 isolates (CrieKom91, 199 and 200) were characterized as possessing KL17 capsular type and O1/O2v1 oligosaccharide type. Four ST395 (CrieKom88, 99, 83 and 68) samples had another capsular type but the same oligosaccharide profile—KL64//O1/O2v1. Capsular and oligosaccharide types for the two ST512 isolates (CrieKom82 and 73) were identified as KL107 and O2/O2v1, correspondingly. It should be noted that *K. pneumoniae* isolates belonging to ST101 and ST512 were collected from oral swabs only, whereas ST395 samples were obtained from both loci (oral swab—CrieKom99 and rectal swab—CrieKom88, 83 and 68).

**Figure 1:**
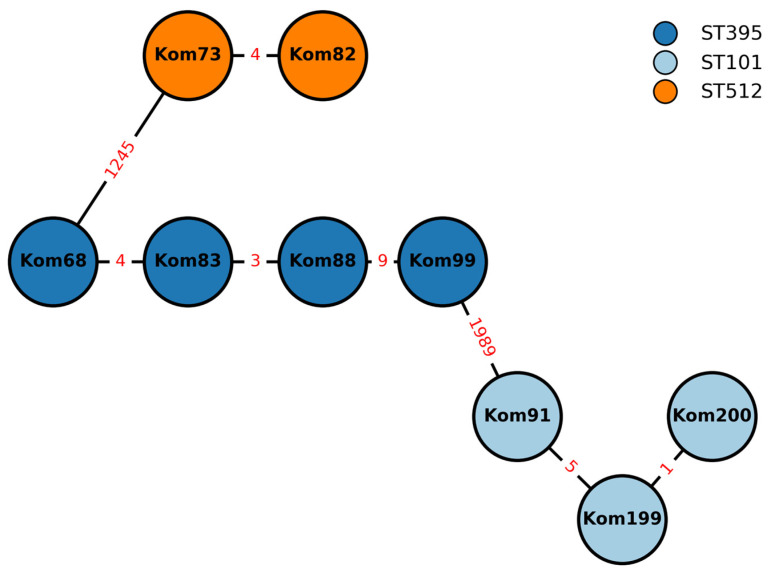
Minimum spanning tree based on cgMLST profiles of clinical *K. pneumoniae* isolates obtained from a single patient. The red values on the edges indicate the number of allele differences between the corresponding isolates. ‘Crie’ prefixes are omitted for simplicity.

CgMLST analysis revealed that whole genome sequences of *K. pneumoniae* isolates were very similar within each lineage. In particular, one and five allele differences were found between oral swab ST101 isolates; two ST512 isolates were characterized by four different cgMLST alleles; rectal and oral swab ST395 isolates differed by nine cgMLST alleles, and 3–4 allele differences were observed within ST395 rectal samples (Figure 1). Thus, according to the criterion described previously (≤18 cgMLST allele differences between the isolates to be considered as belonging to a single strain or clone for *K. pneumoniae* [21]), three ST101, two ST512 and four ST395 isolates were highly likely to represent a single strain/clone within each lineage.

The complete cgMLST profiles of all isolates are presented in Table S1.

We also performed SNP phylogeny analysis for the isolates belonging to ST395 and ST101, respectively. The results are provided in Figures S1 and S2, which reflect essentially the same relationship between the isolates as the cgMLST analysis.

### 2.2. Phenotypic Antimicrobial Resistance (AMR) and AMR Genes

**Figure 2:**
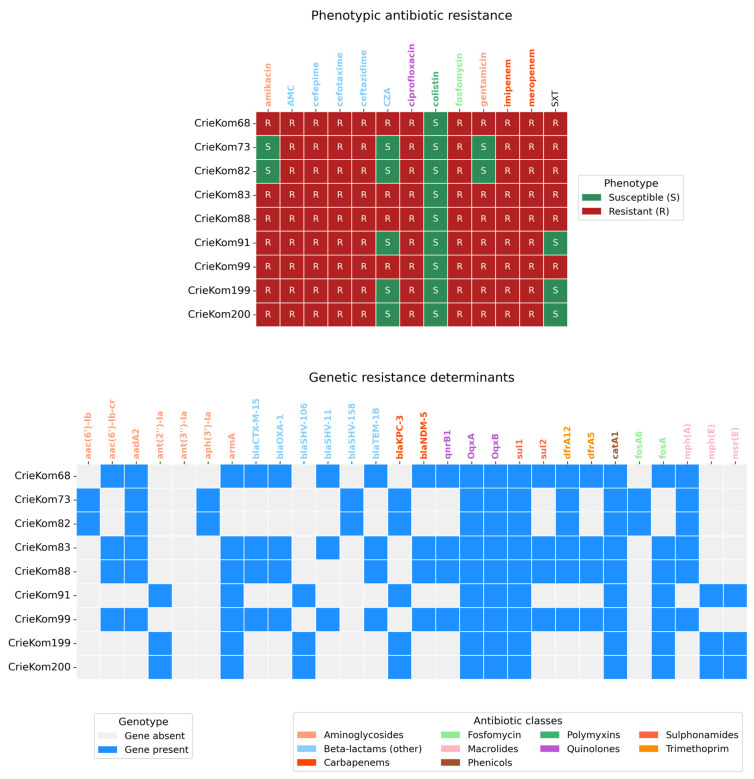
Phenotypic and genomic antimicrobial resistance profiles of clinical *K. pneumoniae* isolates obtained from a single patient. The color scheme links each antibiotic class to its associated resistance genes, with matching colors indicating the relationship. AMC: amoxicillin-clavulanic acid, CZA: ceftazidime-avibactam, SXT: trimethoprim-sulfamethoxazole.

The isolates under investigation showed a MDR phenotype defined as resistance to at least one drug from at least three different antimicrobial classes [22,23]. The resistance to 13 antimicrobial agents representing six antimicrobial classes was determined. The resistance profiles and AMR gene content of the isolates are shown in Figure 2.

Both phenotypic and genotypic resistance profiles were specific to the corresponding STs. All the isolates were resistant to penicillins, carbapenems, fluoroquinolones and cephalosporins (III and IV generations). ST101 and ST512 isolates were susceptible to ceftazidime/avibactam, while ST395 samples isolated both from oral and rectal swabs of the patient exhibited resistance to the mentioned combination drug (Figure 2).

Across all three sequence types, the isolates exhibited a uniform MDR phenotype with resistance to third- and fourth-generation cephalosporins, carbapenems, fluoroquinolones, and trimethoprim-sulfamethoxazole (SXT), while remaining susceptible only to colistin and showing variable susceptibility to amikacin and ceftazidime-avibactam. All isolates carried a shared group of resistance determinants, including the efflux pump genes *oqxA/oqxB*, the extended-spectrum beta-lactamase (ESBL) gene *bla*_CTX-M-15_, *bla*_TEM-1B_, multiple *bla*_SHV_ variants, the quinolone resistance gene *qnrB1*, and sulfonamide and trimethoprim resistance determinants (*sul1/sul2*, *dfrA12/dfrA5*), consistent with the broad non-susceptibility to β-lactams, fluoroquinolones, and SXT.

High-risk carbapenemase genes were unevenly distributed among lineages, with ST395 and ST512 isolates harboring metallo-β-lactamase *bla*_NDM-5_, whereas ST101 strains carried *bla*_KPC-3_ without NDM. Phenotypically, all isolates were resistant to imipenem and meropenem, but only the ST395 clones were resistant to ceftazidime-avibactam, consistent with avibactam’s inactivity against NDM. Aminoglycoside resistance patterns also differed: ST395 and ST101 isolates encoded multiple modifying enzymes including *aac(6′)-Ib-cr* and *aadA2*, together with the 16S rRNA methylase *armA*, correlating with high-level resistance to both amikacin and gentamicin, whereas ST512 isolates lacked *armA* and carried only *aac(6′)-Ib and aph(3′)-Ia*, yielding only low-level resistance to gentamicin and amikacin below the defined MIC threshold.

Phenotypic analysis of the ST101 isolates indicated that these bacteria possibly acquired additional resistance traits during the patient’s stay in the previous hospital. The epidemiological data did not show the presence of ST101 clone in the current ICU during the 2-month period. At the same time, the ST395, which was recovered from both loci during the final week of ICU hospitalization, was previously observed in the current ICU.

### 2.3. Efflux Systems

Bacterial efflux systems play a crucial role in AMR by contributing to intrinsic and acquired AMR in bacteria affecting virtually all classes of antibiotics [24].

In the genomes of all the studied isolates, genes encoding efflux system proteins belonging to different families were identified. The main MDR efflux systems in *Klebsiella* spp., AcrAB and OqxAB, as well as other efflux systems (KpnE, KpnGH, LptD, MacAB, and MsbA), were highly prevalent in our clinical isolates. In addition to contributing to the MDR phenotype, the AcrAB efflux pump may also function as a virulence factor in *K. pneumoniae*, enabling evasion of pulmonary immune defenses and thereby facilitating pneumonia development. [3]. One of the mechanisms of biocide resistance in Gram-negative bacteria is the expression of efflux systems of the SMR family, which are encoded by the *qacE* and *qacEΔ1* genes that were also revealed in all isolates under investigation.

Additionally, all isolates carried OmpA and OmpK37 genes, which determine porin structures that facilitate the transport of substances into the cell. Notably, the expression of porin genes is regulated by the same transcription factors as the efflux pump genes (*ramA*, in particular, was found in all samples of our study), and mutations in them can inhibit porin expression and directly or indirectly cause overexpression of efflux pumps [25,26]. Such mechanisms in Gram-negative bacteria, including *K. pneumoniae*, make them extremely resistant to a wide range of disinfectants and antibiotics.

### 2.4. Virulence Factors

The isolates possessed a variety of virulence factors, including gene clusters encoding adhesive structures (fimbria of I and III types), different iron acquisition systems, genes for immune modulation, type VI secretion system, etc. The complete list of virulence genes revealed is presented in Table S2.

The fimbrial operons *fimA–fimK* (type 1) and *mrkA–mrkJ* (type 3) were present in the genomes investigated with the exception of ST395 isolates, which lacked the *mrkH* gene.

The genomes of the isolates under investigation were characterized by the presence of genes encoding three types of siderophores: aerobactin (*iucABCD* and *iutA*), enterobactin (*ent* gene cluster transported via *fep* genes) and yersiniabactin *(ybt* locus, *irp1*, *irp2* and *fyuA*). Worth noting, the ST512 isolates did not have any genes of yersiniabactin siderophore cluster. In addition, the *rmpA2* gene, which encodes the regulator of the mucoid phenotype, was also found in all isolates except those belonging to ST512.

ST101 isolates differed from the others by absence of some genes conferring immune modulation function. Additionally, ST395 isolates differed in the absence of *impA/tssA* gene representing the Type VI secretion system cluster. Moreover, one sample CrieKom99 within the same ST was characterized by significantly reduced gene composition of the Type VI secretion system cluster (six out of ten genes found in relative isolates).

Kleborate virulence scores [27] were equal to four for ST101 and ST395 isolates, marking the presence of aerobactin and yersiniabactin clusters, and were equal to zero for ST512 isolates, which indicates the absence of apparently functional yersiniabactin, colibactin, or aerobactin clusters.

### 2.5. Plasmids

**Figure 3:**
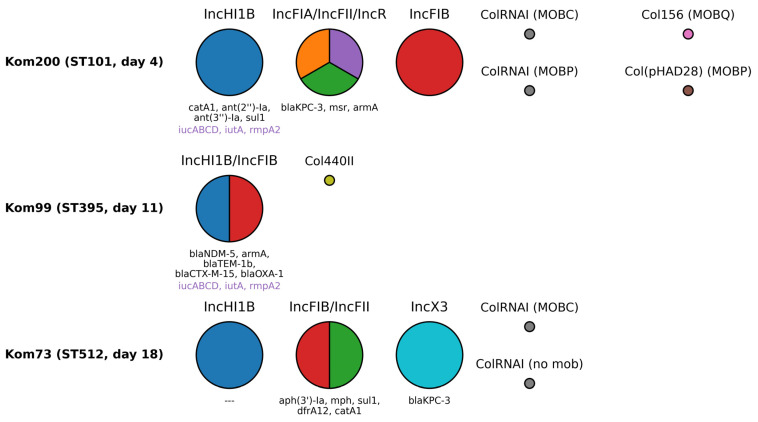
Plasmid content of representative *K. pneumoniae* isolates from a single patient. Plasmid circles are segmented by color to represent multiple replicon types within hybrid plasmids, with each sector corresponding to a distinct incompatibility class. Resistance genes are shown in black, while virulence factors are indicated in magenta below the plasmids containing them. The absence of any resistance or virulence genes in the plasmid is indicated as ‘---‘. Mobility protein types are shown for small plasmids.

All isolates were equipped with several plasmids, which carried a repertoire of AMR and virulence genes. The summary of plasmid content for the representative isolates is provided in Figure 3. The isolates of the same ST had the uniform plasmid content.

Three sequential oral isolates (CrieKom200 (Day 4), CrieKom99 (Day 11), and CrieKom73 (Day 18)) were compared for plasmid replicon content and plasmid-associated resistance and virulence determinants as summarized in Figure 3. Across this longitudinal within-patient series, the data reveal extensive remodeling of the plasmid repertoire, including shifts in dominant incompatibility groups alongside altered distributions of key AMR genes and virulence factors.

The earliest isolate, CrieKom200 (ST101, Day 4), carried a complex plasmid complement dominated by IncHI1B together with an IncFIA/IncFII/IncR multireplicon plasmid, plus additional IncFIB and several small Col-type plasmids (including ColRNAI variants, Col156, and ColpHAD28 having different MOB classes). Functionally, IncHI1B in CrieKom200 was reported to encode a mixed cargo combining AMR genes (*catA1*, *ant(2″)-Ia*, *ant(3′)-Ia*, *sul1*) with a virulence-associated module consisting of aerobactin synthesis/uptake genes (*iucABCD*, *iutA*) and the hypermucoidy regulator *rmpA2*. In the same isolate, the IncFIA/IncFII/IncR plasmid carried *bla*_KPC-3_ together with *armA* and *msr*, while *fosA6* was found to be located on a chromosome.

The intermediate isolate, CrieKom99 (ST395, Day 11), showed a distinct plasmid architecture in which the principal replicon set was reported as IncHI1B/IncFIB, accompanied by an additional small Col440II plasmid. Notably, the IncHI1B/IncFIB plasmid in CrieKom99 combined broad AMR content—including *bla*_NDM-5_, *armA*, *bla*_TEM-1b_, *bla*_CTX-M-15_, and *bla*_OXA-1_—with the same virulence-associated loci (*iucABCD*, *iutA*, *rmpA2*) that were present in CrieKom200. Thus, between days 4 and 11, virulence potential linked to aerobactin and *rmpA2* was retained at the plasmid level despite a change in clonal background (ST101 to ST395) and a shift in the principal plasmid scaffold carrying these determinants.

The late isolate, CrieKom73 (ST512, day 18), again differed in plasmid composition, with IncHI1B, IncFIB/IncFII, and IncX3 listed among the main plasmids and additional ColRNAI replicons reported (including a “MOBC” and a “no MOB” variant). In contrast to CrieKom200 and CrieKom99, the IncHI1B plasmid in CrieKom73 was annotated as carrying no identified resistance or virulence genes, while *bla*_KPC-3_ was associated with IncX3, and a separate hybrid plasmid encoded multiple non-β-lactam resistance genes (*aph(3′)-Ia*, *mph*, *sul1*, *dfrA12*, *catA1*). Importantly, no plasmid-associated *iucABCD*/*iutA*/*rmpA2* loci were reported for CrieKom73, suggesting loss (or at least non-detection/absence) of the aerobactin–*rmpA2* virulence module by day 18.

## 3. Discussion

In this study, we deciphered the genetic diversity of isolates obtained from oral and rectal swabs of a single patient staying three weeks in ICU using WGS and a set of bioinformatics analyses. Few studies, to date, have focused on analyzing *K. pneumoniae* isolates collected from individual patients, while most clinical research has instead involved comparisons between isolates derived from separate individuals. In contrast to inter-patient studies, longitudinal intra-patient investigations enable the detection of clinically relevant microevolution events, including plasmid rearrangements and the emergence of adaptive mutations in resistance, virulence, and capsule synthesis genes, as well as the stepwise accumulation of these mutations and their dynamic changes that facilitate host-specific adaptation and selection of niche-adapted subpopulations [28]. In addition, intra-patient studies can reveal possible changes within bacterial population, namely, the substitution of a strain/clone with another one or their co-existence during some period. Our investigation focused on nine CRKP isolates obtained during intensive care treatment of a single patient.

The isolates obtained during the first week of the ICU stay represented the first strain that was assigned to ST101 and CG101. Since the first ST101 isolate was revealed less than 48 h upon ICU admission, it was likely acquired during a prior patient hospitalization. Notably, ST101 is a high-risk clone previously reported to combine virulence and resistance traits, which was especially prevalent in Europe and several countries from other parts of the world [7,29]. The ST101 lineage emerged quite recently and contributed to dissemination of CRKP causing infections with higher mortality and morbidity rates [7,30]. All ST101 isolates studied here were MDR, and contained both carbapenemase *bla*_KPC-3_ and ESBL *bla*_SHV-106_ genes. The *bla*_KPC-3_ gene represents one of the most prevalent *bla*_KPC_ variants among *Enterobacteriaceae* species and is typically located on plasmids harboring the Tn4401 transposon, which facilitates its widespread dissemination [31]. The ESBL gene *bla*_SHV-106_ emerged relatively recently, exhibited a restricted distribution, and was associated with the IS26 insertion element [32,33].

The second lineage comprised one oral and three rectal isolates belonging to ST395 and CG258. Given that these samples were obtained 11–18 days after ICU admission and were absent earlier, ST395 isolates likely originated from the clones circulating within the ICU. These four isolates were MDR and exhibited high resistance to ceftazidime-avibactam, carbapenems and extended-spectrum cephalosporins due to the presence of *bla*_NDM-5_ and *bla*_CTX-M-15_. ST395 isolates retained high virulence owing to the same virulence factors as ST101. It should be mentioned that *K. pneumoniae* ST395 represents a high-risk clonal lineage that induced local outbreaks and multiple sporadic cases in Russia, some other European countries, Kazakhstan and USA [34].

The third genetic lineage comprised two oral isolates (CrieKom73 and CrieKom82) recovered on the 14th and 18th day of hospitalization, respectively. These isolates were identified as ST512 also belonging to CG258. We hypothesized that ST512 clones represented additional, hospital-acquired variants co-circulating with ST395. These MDR isolates were highly resistant to carbapenems also due to the *bla*_KPC-3_ gene. Several studies have demonstrated that ST512 is one of the best-characterized MDR clones of *K. pneumoniae*, frequently colonizing and infecting individuals with persistent illness [35,36]. The ST512 epidemic clone warrants particular concern given its global spread and consistent carbapenemase production [37,38]. Furthermore, ST512 showed particular predominance in ICU, where intensive antimicrobial drug use and rapid patient turnover created ideal conditions for its high transmissibility and persistence [37].

ST395 and ST512 belong to CG258, which is globally disseminated and is associated with outbreaks of CRKP due to high pathogenicity and transmissibility combined with increased duration of colonization [27,39]. For this reason, *K. pneumoniae* CG258 is intensively studied in many countries, but only a few works describe the diversity of isolates within a single patient. For example, longitudinal research of one French patient who was colonized by *K. pneumoniae* for 4.5 years revealed 17 isolates belonging to CG258 [40]. Similar findings were obtained in one Colombian hospital during the investigation of intra-patient genetic diversity [31]. The recovered isolates harbored *bla*_KPC_ carbapenemase gene and were assigned to three different STs, all of which belonged to the same pandemic CG258 [31]. In China, four hv-CRKP isolates belonging to ST11 (CG258) were isolated from an 86-year-old patient with severe pneumonia during a two-week stay in hospital [41].

These findings contrast with our results, in which the nine recovered isolates were clustered into two distinct clonal groups (CG101 and CG258). 

Furthermore, in Argentina a high-risk clone of *K. pneumoniae* belonging to MDR ST258/CG258 colonized a 66-year-old female with ovarian cancer. During hospitalization, it changed first to carbapenemase-producing *E. coli* (ST370) and then again to ST11/CG258 CRKP [42]. Finally, a large-scale investigation of ESBL-producing Enterobacterales in Switzerland revealed that 84.9% of patients were colonized by only one cgMLST cluster of ESBL-PE [20].

Another longitudinal intra-patient observation demonstrated that all *K. pneumoniae* isolates recovered from various loci in three patients belonged to a single cgMLST cluster. Overall, approximately 20% of consecutive patients harbored a single *K. pneumoniae* strain, whereas roughly 5% of patients were colonized with multiple bacterial strains [20].

Notably, the nine *K. pneumoniae* isolates analyzed by us demonstrated unusual diversity, spanning three different STs grouped into two separate CGs—a pattern rarely observed in clinical isolates obtained from a single unit or patient.

The temporal dynamics of within-host *K. pneumoniae* colonization necessitates differential interpretation of isolates recovered from distinct loci, particularly when clinical infection is confirmed. In the context of viral-bacterial pneumonia, respiratory tract samples demonstrate superior clinical relevance in distinguishing active pathogenic involvement from mere colonization compared to fecal swabs [43,44]. In the patient under study, sequential *K. pneumoniae* clones isolated from oral swabs exhibited a marked shift from ST101 (days 1–7) to ST395 (day 11) and subsequently to ST512 (days 14 and 18), representing a stepwise clonal replacement pattern. The concurrent epidemiological surveillance documented the presence of ST395 and ST512 in other ICU patients during the study period, whereas ST101 was not recovered from any other hospitalized individuals, suggesting acquisition of the initial clone prior to ICU admission and subsequent replacement by dominant circulating ICU clones through cross-transmission or environmental exposure.

Plasmid analysis provided useful insights into the mechanisms involved in bacterial resistance and virulence transmission. Taken together, the plasmid content data support the suggestion of a transition from isolates with both high-risk resistance (carbapenemases such as *bla*_KPC-3_ and *bla*_NDM-5_) and a plasmid-borne hypervirulence-associated signature (*iucABCD*/*iutA*/*rmpA2*) toward the later isolates in which resistance determinants persist but the virulence-associated plasmid module is no longer present in the reported plasmid gene content. In this interpretation, plasmids appear to play a central mechanistic role in the within-patient shift from “more dangerous” (convergence of extensive AMR and aerobactin/*rmpA2* on IncHI1B-family plasmids) to “less virulent” (maintenance of AMR, including *bla*_KPC-3_ on IncX3, coupled with apparent loss of the aerobactin/*rmpA2* module). This conclusion reflects genomic virulence potential inferred from the reported gene repertoire rather than a direct phenotypic measurement of virulence. However, the plasmid composition may reflect lineage-specific characteristics of the isolates rather than genuine horizontal gene and transposon transfer events.

The apparent reduction, according to genomic data, in virulence-associated features in the terminal clones warrants particular attention. The ST512 isolates (CrieKom73 and CrieKom82) demonstrated a substantially diminished virulence potential and were found to be deficient in *bla*_NDM_ carbapenemase genes, in contrast to the ST395 isolate, which retained this resistance determinant. Previous reports also revealed higher virulence factor content for ST395 and ST101 isolates than for ST512 [45,46]. In the present study, this finding is consistent with the hypothesis that there has been selective pressure exerted by broad-spectrum antimicrobial therapy, which may be indicative of preferential suppression of highly resistant variants. In contrast, the persistent isolation of ST395 from rectal swabs on days 11, 14, and 18 indicates the establishment of gastrointestinal colonization with an ICU-prevalent clone. However, the absence of clinical manifestations of invasive gastrointestinal infection and the isolation of less virulent clones from the primary infection locus (respiratory tract) suggest compartmentalized colonization rather than generalized systemic infection driven by this clone.

It is acknowledged that the utilization of a limited number of *K. pneumoniae* isolates obtained from a single patient imposes a limitation on the capacity to accurately monitor the transmission of AMR bacteria among patients and within a healthcare facility. Meanwhile, we can suggest that isolates obtained from the series of rectal swabs corresponded to *K. pneumoniae* lineages circulating within a specific medical unit. At the same time, oral swab isolates were identified as the “strain-winner” in host–bacteria interactions, thereby successfully colonizing and subsequently infecting the patient.

## 4. Materials and Methods

### 4.1. Case Report

A 44-year-old male patient was transferred from another hospital with a primary diagnosis of coronavirus infection, confirmed by a positive PCR test for SARS-CoV-2. The previous hospitalization was from 28 August 2021 until 9 September 2021, and on the third day after admission (30 August 2021) the patient received a single-dose intravenous infusion of tocilizumab (800 mg). The patient then was transferred to the ICU of a clinic specializing in the treatment of COVID-19 patients. He was connected to mechanical ventilation from the first day of admission to the ICU (9 September 2021). Moreover, the main diagnosis was complicated by community-acquired bilateral polysegmental viral-bacterial pneumonia of severe course, bilateral pneumothorax, multiple organ failure (respiratory, cerebral), sepsis and thrombosis of the labial veins of the right lower limb with flotation. Concomitant diseases included catheter-associated urethritis and chronic maxillary odontogenic right-sided sinusitis. In addition, the patient was obese (body mass index equal to 33.2 kg/m^2^).

**Figure 4:**
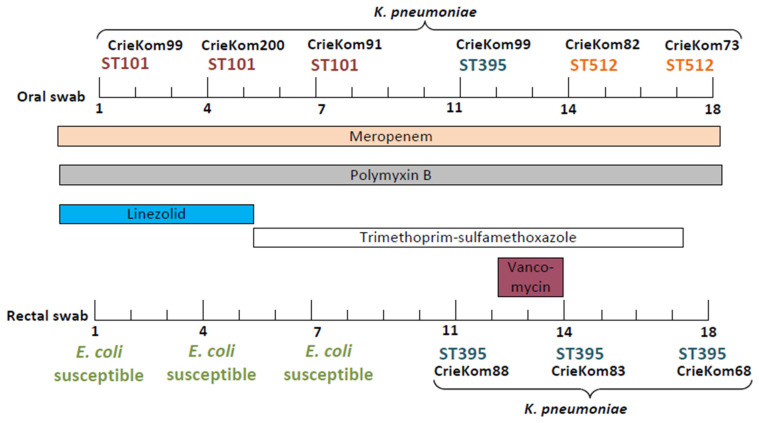
The course of bacterial colonization and prescribed antimicrobial therapy for the ICU patient. Isolate names and sequence types (ST) are indicated near the date of their isolation. The numbers on the timeline indicate the days since patient admission.

The prescribed antimicrobial therapy (Figure 4) included meropenem (3 g per day, for 21 days), polymyxin B (200 mg per day, for 21 days) and linezolid (1200 mg per day, for 6 days) starting from the first day of ICU hospitalization. On the sixth day of admission, linezolid was replaced with trimethoprim-sulfamethoxazole (100 mg per day, for 13 days) and vancomycin (4 g, once per day) was administered twice on the 14th and 15th days. Additionally, an antiviral drug (remdesivir, 100 mg per day, for 21 days), an anti-inflammatory and immunosuppressant agent (dexamethasone, 16 mg per day, for 14 days), anticoagulants (enoxaparin or heparin, 160 mg per day for 6 days, then 240 mg per day for 8 additional days) and a proton pump inhibitor (Omeprasole, 40 mg per day, for 17 days) supplemented the antimicrobial therapy. The patient remained in the ICU for three weeks and, following clinical improvement, was transferred to the internal medicine department, where he spent an additional three weeks receiving Remdesivir (100 mg per day) and Omeprazole (40 mg per day) as supportive therapy every day.

The next day after admission to the ICU (10 September 2021) and every third day of patient hospitalization in this department, oral and rectal swabs were taken and analyzed to determine the colonizing bacteria. Twelve bacterial cultures obtained were identified to species level by microbiological methods and tested for antibiotic susceptibility. Bacterial isolates exhibiting MDR to antimicrobial compounds were subjected to WGS. Over the three-week treatment and follow-up period in the ICU, three *Escherichia coli* and nine *K. pneumoniae* isolates were recovered from oral and rectal swabs. *E. coli* isolates obtained from rectal samples were excluded from subsequent analyses because they were susceptible to all tested antimicrobial agents.

### 4.2. Sample Acquisition, Susceptibility Testing and DNA Isolation

Samples collected from each locus were cultured using a semi-quantitative sectoral approach on the following media: Columbia blood agar base and Urinary Tract Infections Chromogenic Agar (Thermo Fisher Scientific, Waltham, MA, USA). Cultivation proceeded according to standard clinical microbiology protocols, with inocula incubated at 36 °C for 18–24 h. Following incubation, bacterial growth was assessed, enumerated in CFU/ml, and preliminarily identified. Subsequent isolation on selective Endo agar (Thermo Fisher Scientific, Waltham, MA, USA) yielded isolates that were identified to species level and subjected to antimicrobial resistance profiling.

Species identification of all isolates was performed by matrix-assisted laser desorption/ionization time-of-flight mass spectrometry (MALDI-TOF MS) using a MALDI Biotyper Microflex LT/SH system (Bruker, Karlsruhe, Germany). Antimicrobial susceptibility was assessed through disk diffusion testing on Mueller–Hinton agar plates (Gem, Moscow, Russia) inoculated with antibiotic-impregnated disks (BioRad, Marnes-la-Coquette, France), supplemented by broth microdilution using a VITEK 2 Compact 30 analyzer (bioMérieux, Marcy-l’Étoile, France) for minimum inhibitory concentration (MIC) determination. The antibiotic panel included the following drugs: amikacin, amoxicillin-clavulanic acid (AMC), cefepime, cefotaxime, ceftazidime, ceftazidime-avibactam (CZA), ciprofloxacin, colistin, fosfomycin, gentamicin, imipenem, meropenem, trimethoprim-sulfamethoxazole (SXT).

The results of antimicrobial susceptibility testing were interpreted in accordance with the criteria of European Committee on Antimicrobial Susceptibility Testing (EUCAST) version v 11.0 (http://www.eucast.org, accessed on 25 October 2025).

Genomic DNA was isolated from the bacterial cultures using a DNeasy Blood and Tissue Kit (Qiagen, Hilden, Germany). The obtained DNA was subsequently used to generate sequencing libraries following two complementary approaches: short-read libraries prepared with the Nextera™ DNA Sample Preparation Kit (Illumina^®^, San Diego, CA, USA) and, for selected representative isolates, long-read libraries employing the Rapid Barcoding Sequencing Kit SQK-RBK004 (Oxford Nanopore Technologies, Oxford, UK).

### 4.3. WGS and Data Analysis

The sequencing was conducted on a NextSeq platform (Illumina^®^, San Diego, CA, USA) for high-throughput short-read sequencing and a MinION device (Oxford Nanopore Technologies, Oxford, UK) for long-read sequencing.

Read quality control was performed employing flexbar 3.5 [47], which implemented the following filtering parameters: a minimum quality score threshold of Q20, quality-based trimming at read termini, elimination of sequencing adapter sequences, exclusion of homopolymeric A/T regions, and removal of fragments shorter than 60 bp.

Base identification from raw MinION sequencing data was performed using Guppy Basecalling Software version 6.4.6 (Oxford Nanopore Technologies, Oxford, UK), followed by sample demultiplexing via Guppy Barcoding Software version 6.4.6 (Oxford Nanopore Technologies, Oxford, UK). Reads shorter than 1000 bp were excluded from downstream analysis.

Short-read sequence assemblies were generated using SPAdes version 3.15.4 (--isolate mode) [48], with contigs shorter than 500 bp being removed. Hybrid assemblies were constructed utilizing Unicycler version 0.5.0 (--normal mode) [49], with a minimum contig length threshold of 1000 bp.

Assembly quality was assessed using Quast version 5.3.0 (https://github.com/ablab/quast, accessed on 29 October 2025). The presence of conserved genes in the assembled contigs was evaluated using BUSCO version 6.1.0 (https://busco.ezlab.org/, accessed on 25 May 2026) using the enterobacterales_odb12.2 lineage dataset.

Multilocus sequence typing (MLST) was performed using the Institut Pasteur database (https://bigsdb.pasteur.fr/cgi-bin/bigsdb/bigsdb.pl?db=pubmlst_klebsiella_seqdef, accessed on 12 October 2025) using the 7-loci typing scheme described in [50].

Capsular polysaccharide (KL) and lipooligosaccharide (O) loci were identified using Kaptive v. 3.1.0 [51] with default settings (database version dated 14 October 2025).

Core genome MLST (cgMLST) profiles were generated using MentaLiST software [52] (https://github.com/WGS-TB/MentaLiST, version 0.2.4, accessed on 21 October 2025) implementing the 2358 loci scheme obtained from cgmlst.org (https://www.cgmlst.org/ncs/schema/schema/2187931/, accessed on 21 October 2025) with default parameters.

The AMR genes were revealed by means of Resfinder 4.6.0 [53] (http://genepi.food.dtu.dk/resfinder, accessed on 10 October 2025, using default parameters). Virulence factor identification within *K. pneumoniae* genomes was conducted via the Virulence Factor Database (VFDB) [54] (http://www.mgc.ac.cn/VFs/main.htm, accessed on 16 October 2025) employing standard parameters. We also used Kleborate version 3.2.4 (default parameters) [27] to quantitatively compare the virulence and AMR potential of the isolates.

Plasmid replicon typing was performed by PlasmidFinder 2.1 with default parameters (https://cge.food.dtu.dk/services/PlasmidFinder/, accessed on 28 October 2025) and additionally verified using mob_typer v. 3.1.9 from MOB-suite package [55] with default parameters. Plasmid assembly was facilitated by mlplasmids [56] with default parameters and mob_recon v. 3.1.9 [55].

Supplementary data manipulation and result formatting were accomplished using a custom computational pipeline developed in our previous studies [2,57].

## 5. Conclusions

All isolates recovered from a single ICU patient belonged to high-risk, multidrug-resistant *Klebsiella pneumoniae* clonal lineages, but differed in inferred virulence potential. The detection of carbapenemase genes (*bla*_KPC-3_ and *bla*_NDM-5_) in conjunction with a broad repertoire of virulence-associated factors, underscores the need for rigorous infection prevention and control measures, in addition to sustained genomic surveillance, to curtail the dissemination in healthcare settings.

A within-host shift in oral-swab isolates, with a greater propensity to be associated with subsequent infection, has been observed. This shift indicates a transition from the virulent ST101 and ST395 lineages to the comparatively less virulent, according to the genomic data, ST512 lineage, which shows the possibility of antibiotic-driven selection under intensive therapy (meropenem plus polymyxin B). This hypothesis is further substantiated by the presence of lineage-specific disparities in plasmid content, which are indicative of differential retention or acquisition of mobile genetic elements in response to drug pressure. However, additional plasmid transfer experiments are required to verify such selection process.

Further research is necessary to elucidate the epidemiology and transmission dynamics of these strains, thereby facilitating more effective public health interventions. The co-occurrence and turnover of multiple resistant lineages within a single patient represents an emerging challenge in hospital epidemiology and argues for surveillance approaches that explicitly account for dynamic within-host evolution of carbapenem-resistant *K. pneumoniae* during prolonged hospitalization.

## Figures and Tables

**Table 1:** Genome assembly properties and typing results for clinical *K. pneumoniae* isolates studied.

Isolate ID	ST	Average Coverage	Assembly Type	Number of Contigs	Genome Total Length (Including Plasmids), Mbp	Completeness (BUSCO Score)	GC Content
CrieKom68	ST395	125×	Short-read	118	5.79	99.2%	0.567
CrieKom73	ST512	117×	Hybrid	7	5.84	99.7%	0.568
CrieKom82	ST512	50×	Hybrid	9	5.89	99.6%	0.567
CrieKom83	ST395	53×	Short-read	151	5.81	99.1%	0.567
CrieKom88	ST395	90×	Short-read	131	5.80	99.2%	0.567
CrieKom91	ST101	76×	Hybrid	10	6.12	99.8%	0.564
CrieKom99	ST395	43×	Hybrid	8	5.84	99.8%	0.566
CrieKom199	ST101	49×	Short-read	128	6.09	99.2%	0.564
CrieKom200	ST101	73×	Hybrid	10	6.11	99.4%	0.564

## Data Availability

The original contributions presented in the study are included in the article/Supplementary Material. Genomic data for all isolates was deposited to the public database Genbank (https://www.ncbi.nlm.nih.gov/genbank/, accessed on 10 June 2026) under the project PRJNA1426063.

## Supplementary Materials

The following supporting information can be downloaded at: https://www.mdpi.com/article/10.3390/antibiotics15060605/s1. Table S1: cgMLST profiles of the *K. pneumoniae* isolates obtained from a single patient, Table S2: The complete list of virulence genes revealed in the clinical *K. pneumoniae* isolates obtained from a single patient, Figure S1: SNP-based tree for the isolates of ST395, Figure S2: SNP-based tree for the isolates of ST101.
