# Supplementary figures and images for: Short-Term Within-Host Genomic Diversity and Clone Turnover of Carbapenem-Resistant Klebsiella pneumoniae in an Intensive Care Unit Patient
Source: Antibiotics (Basel). 2026 Jun 14;15(6):605. doi: 10.3390/antibiotics15060605 (PMC13296264; doi:10.3390/antibiotics15060605)

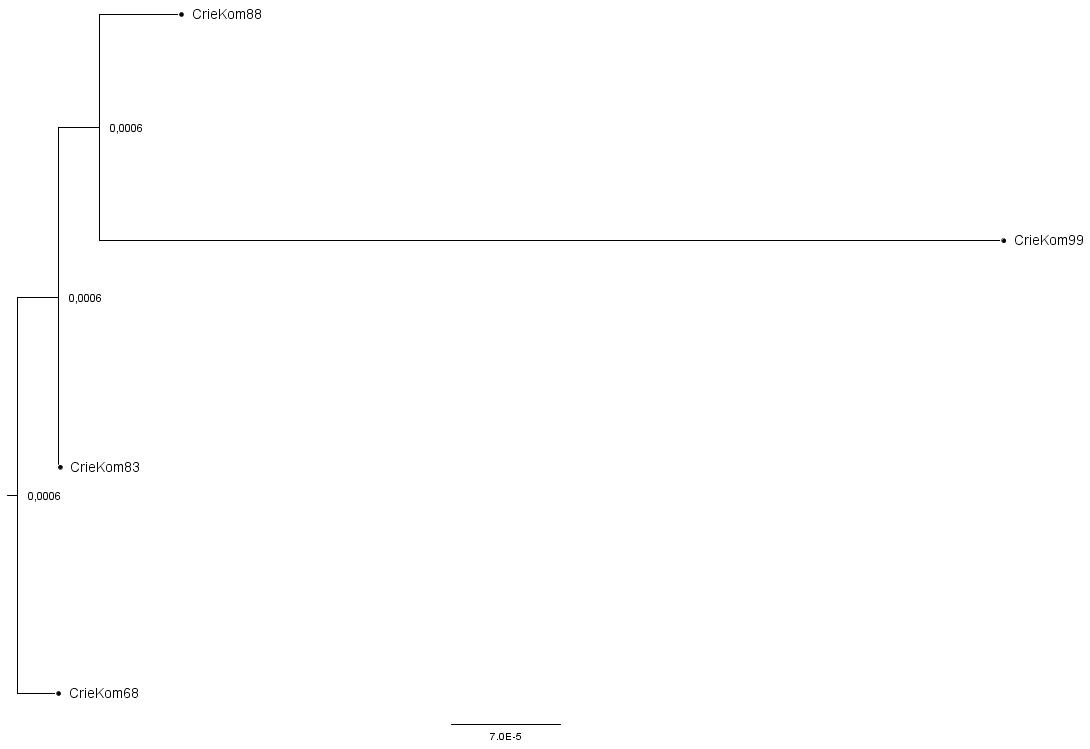

Supplement: Supplementary file 1 [file antibiotics-15-00605-s001.zip › FigS1.jpg]

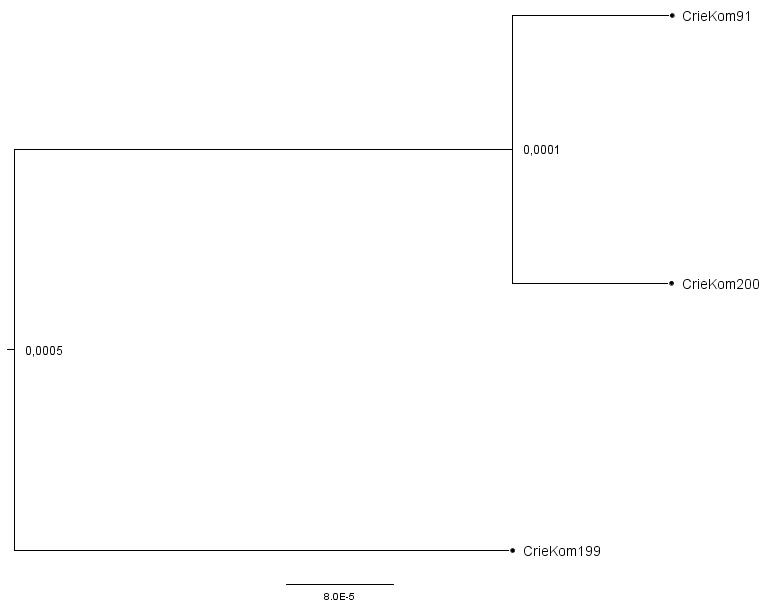

Supplement: Supplementary file 1 [file antibiotics-15-00605-s001.zip › FigS2.jpg]
